# Social determinants, their relationship with leprosy risk and temporal trends in a tri-border region in Latin America

**DOI:** 10.1371/journal.pntd.0006407

**Published:** 2018-04-06

**Authors:** Ivaneliza Simionato de Assis, Marcos Augusto Moraes Arcoverde, Antônio Carlos Viera Ramos, Luana Seles Alves, Thais Zamboni Berra, Luiz Henrique Arroyo, Ana Angélica Rêgo de Queiroz, Danielle Talita dos Santos, Aylana de Souza Belchior, Josilene Dália Alves, Flávia Meneguetti Pieri, Reinaldo Antônio Silva-Sobrinho, Ione Carvalho Pinto, Clodis Maria Tavares, Mellina Yamamura, Marco Andrey Cipriani Frade, Pedro Fredemir Palha, Francisco Chiaravalloti-Neto, Ricardo Alexandre Arcêncio

**Affiliations:** 1 Graduate Program in Public Health Nursing, Nursing College of Ribeirão Preto, University of São Paulo, Ribeirão Preto, São Paulo, Brazil; 2 Graduate Program Interunit Doctoral Program in Nursing, University of São Paulo at Ribeirão Preto College of Nursing, Ribeirão Preto, São Paulo, Brazil; 3 Secretary of State for Public Health, Natal, Rio Grande do Norte, Brazil; 4 Department of Nursing, Universidade Estadual de Londrina, Londrina, Paraná, Brazil; 5 Graduate Program in Public Health in Border Region, State University of West of Paraná, Foz do Iguaçu, Paraná, Brazil; 6 Department of School of Nursing and Pharmacy, Federal University of Alagoas, Maceió, Alagoas, Brazil; 7 Division of Dermatology of the Department of Internal Medicine of the Ribeirão Preto Medical School, University of São Paulo, Ribeirão Preto, São Paulo, Brazil; 8 Department of Epidemiology, School of Public Health, University of São Paulo, São Paulo, Brazil; Hospital Infantil de Mexico Federico Gomez, UNITED STATES

## Abstract

**Background:**

Brazil is the only country in Latin America that has adopted a national health system. This causes differences in access to health among Latin American countries and induces noticeable migration to Brazilian regions to seek healthcare. This phenomenon has led to difficulties in the control and elimination of diseases related to poverty, such as leprosy. The aim of this study was to evaluate social determinants and their relationship with the risk of leprosy, as well as to examine the temporal trend of its occurrence in a Brazilian municipality located on the tri-border area between Brazil, Paraguay and Argentina.

**Methods:**

This ecological study investigated newly-diagnosed cases of leprosy between 2003 and 2015. Exploratory analysis of the data was performed through descriptive statistics. For spatial analysis, geocoding of the data was performed using spatial scan statistic techniques to obtain the Relative Risk (RR) for each census tract, with their respective 95% confidence intervals calculated. The Bivariate Moran I test, Ordinary Least Squares (OLS) and Geographically Weighted Regression (GWR) models were applied to analyze the spatial relationships of social determinants and leprosy risk. The temporal trend of the annual coefficient of new cases was obtained through the Prais-Winsten regression. A standard error of 5% was considered statistically significant (*p* < 0.05).

**Results:**

Of the 840 new cases identified in the study, there was a predominance of females (n = 427, 50.8%), of white race/color (n = 685, 81.6%), age range 15 to 59 years (n = 624, 74.3%), and incomplete elementary education (n = 504, 60.0%). The results obtained from multivariate analysis revealed that the proportion of households with monthly nominal household income per capita greater than 1 minimum wage (β = 0.025, p = 0.036) and people of brown race (β = -0.101, p = 0.024) were statistically-significantly associated with risk of illness due to leprosy. These results also confirmed that social determinants and risk of leprosy were significantly spatially non-stationary. Regarding the temporal trend, a decrease of 4% (95% CI [-0.053, -0.033], *p* = 0.000) per year was observed in the rate of detection of new cases of leprosy.

**Conclusion:**

The social determinants income and race/color were associated with the risk of leprosy. The study’s highlighting of these social determinants can contribute to the development of public policies directed toward the elimination of leprosy in the border region.

## Author summary

The World Health Organization considers Brazil to be a priority country for the control of leprosy. This is determined by the high rates of prevalence, detection of new cases and proportion of cases in females, proportion of cases in children, and people with degree of incapacity II. Moreover, Brazil has been unable to achieve the target of one case per 10,000 inhabitants, despite efforts to eliminate the disease. Elimination and control become even more complex challenges in border regions if we consider the heavy flow of people between countries and the resulting overload for the local health system. Thus, this study aimed to identify the risk of leprosy and its social determinants, as well as to investigate the temporal trend of the disease in a region with a triple border, connecting Brazil, Paraguay and Argentina. We identified 840 new cases in the study; there was a predominance of women (n = 427, 50.8%), of white race/color (n = 685, 81.6%), age range 15 to 59 years (n = 624, 74.3%), with incomplete elementary education (n = 504, 60.0%). The social determinants income and race/color were associated with the risk of leprosy. The results may contribute to the planning of actions in health for border regions.

## Introduction

Leprosy is a chronic, disabling infectious disease caused by *Mycobacterium leprae*, an intracellular bacillus that attacks Schwann cells (cells of the peripheral nervous system) causing their destruction and leading to severe neuropathies that cause deformities and physical disabilities [1–4]. Leprosy, an endemic member of the neglected diseases group [5], mainly affects developing countries, constituting a serious global public health problem.

In 2015, the global leprosy detection rate was 3.2 cases per 100,000 inhabitants, with 16 countries accounting for 92% of all cases of the disease. India, Brazil and Indonesia reported more than 10,000 cases, comprising 81% of new cases of the disease during the period [6]. Brazil is the country with the second highest number of cases (surpassed only by India), with a detection rate of 14.06 cases per 100,000 inhabitants in 2015 [7].

The World Health Organization (WHO) has launched the Global Leprosy Strategy 2016–2020, which aims “Accelerating towards a leprosy-free world”, which aims to detect leprosy early and provide immediate treatment to prevent disability and reduce transmission of the disease in the community [8]. This strategy aims to reduce the prevalence of leprosy by improving the capacity of health services to diagnose cases in the early stages of the disease, to provide timely treatment aimed at a cure, and to eliminate sources of infection [9].

One of the main obstacles in the elimination of leprosy concerns the social inequalities faced by people in developing countries, caused by poor housing conditions, low levels of education, low incomes, deficits in health services, and migration to urban centers [10,11].

Endemic areas with unhealthy housing conditions lacking basic sanitation, human agglomeration, the sharing of collective spaces, especially dormitories with shared beds and sleeping nets, are risk factors for the transmissibility and development of the disease [12,13].

So, understanding the social determinants that influence the risk of leprosy transmission is of fundamental importance for the development of actions and strategies that aim to accelerate the process of elimination of the disease in Brazil [14].

For the WHO [14] social determinants of health include individual behaviors and living and working conditions, as well as the relationship between the economic, cultural and social structure. Chaptini & Marshman, based on this premise, grouped social determinants into three dimensions, these being socioeconomic, environmental, and gender determinants [10].

A review of the literature, using the descriptors “social determinants” and “leprosy”, found few studies with this focus in Brazil. Among the studies that proposed to test the relationship between social determinants and leprosy risk [15–18], none were found that considered spatial dependence or incorporated it into their analysis.

By definition, spatial dependence is understood to be the tendency that the value of a variable associated with a particular location resembles the value of its neighboring samples more than the rest of the locations of the sample set; it also becomes the concept of neighborhood, which is formulated on the basis of the property of proximity, in which the events that are closer together tend to be more similar to each other than distant events [19].

Thus, a case of leprosy in a given region tends to influence the epidemiological situation of its neighbors, therefore not being a random event. The quantification and appropriate use of methodologies to deal with this characteristic are also considered to be a knowledge gap, since the majority of traditional epidemiological studies tend to ignore this effect.

There is also a lack of studies in border areas of Brazil, where these diseases tend to be more critical or more difficult to control due to the fragility of the services in monitoring the dynamics or migration of populations between the different countries [20]. Therefore, this study aimed to analyze social determinants and their relationship to the risk of contracting illness due to leprosy, as well as the temporal trends of leprosy cases in a tri-border region in Latin America.

## Methods

### Study design

This was an ecological study [13].

### Scenario

**Fig 1:**
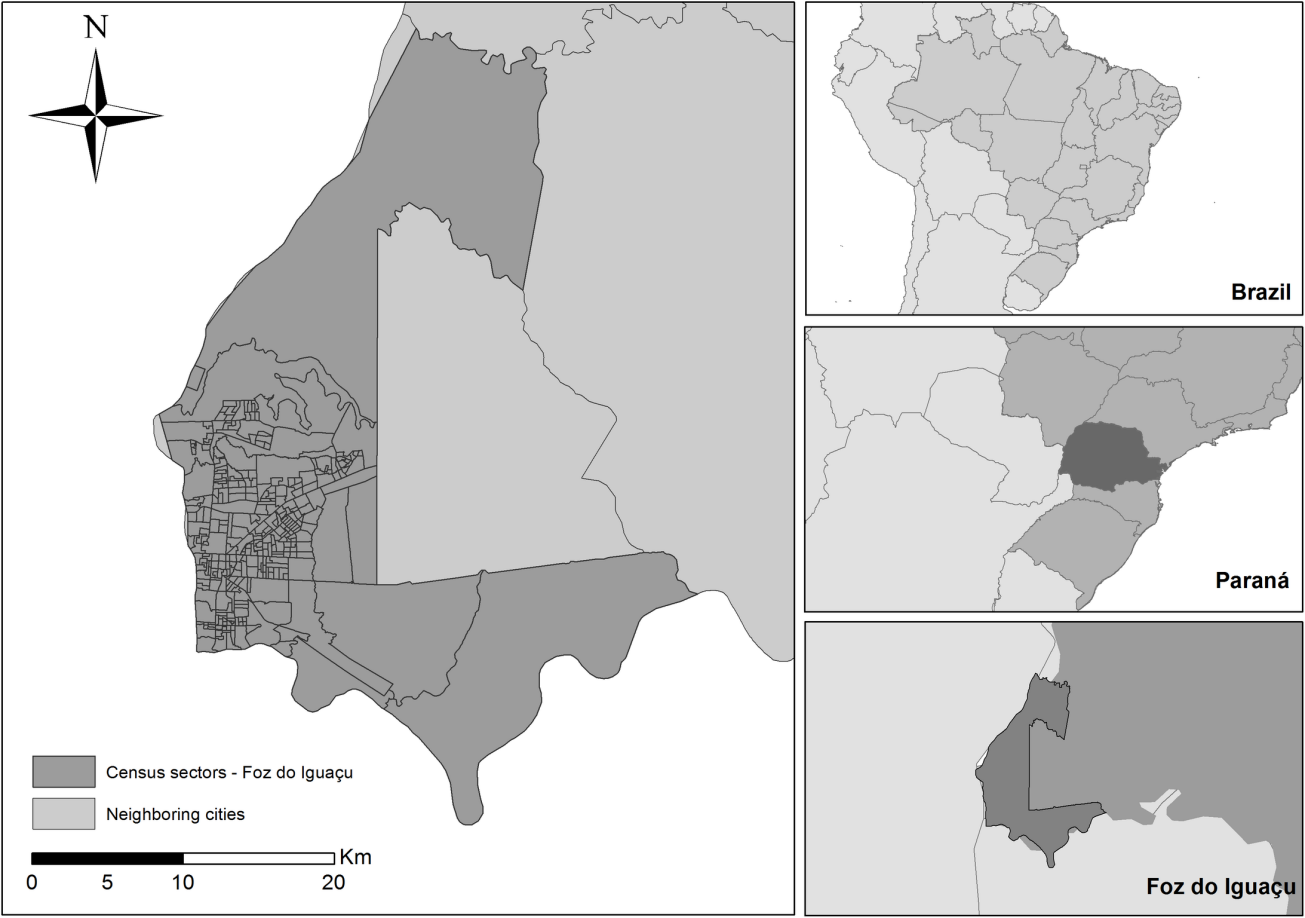
Geographic location of the tri-border region of Brazil, Paraguay and Argentina.

The municipality of Foz do Iguaçu, is on the triple border between Brazil, Paraguay, and Argentina (Fig 1), situated in the extreme west of Paraná, it has a total area of 618,353 km^2^, being the seventh most populous city in the state. It is organized in 327 census tracts, 320 in the urban area and seven in the rural area. The population of the municipality of Foz do Iguaçu is approximately 263,915 inhabitants, with 99.2% of the population residing in the urban area [21].

Table 1 presents the main socioeconomic information regarding the municipality. It reveals important social indicators, such as an equality of income distribution of 0.545, which represents an unequal income distribution, and a human development index of 0.751, close to the national average. A critical social condition observed was that only 75.3% of the households had an adequate sewage system [21], which constitutes a risk factor for leprosy [10].

**Table 1:** Socioeconomic information for the municipality of Foz do Iguaçu, Paraná, Brazil.

Indicators	Values
Human Development Index (HDI)	0.751
Gini Index	0.545
Subjective poverty index	25.5%
Unemployment rate	7.05%
Population with incomes less than ½ minimum wage	25.5%
Average salary of formal workers	2.7 minimum wages
Illiteracy rate (≥ 15 years of age)	5.64%
Households with adequate sewage system	75.3%
Average number of residents per household	3 residents

Source: IBGE (2010)

In the context of healthcare, Foz do Iguaçu has 388 health facilities; 61 public, 321 private, and six non-profit institutions. The Municipality has 30 Primary Health Units (some with a Family Health Strategy), 25 specialized clinics/outpatient clinics, two emergency care units, three psychosocial care centers, 46 diagnostic and therapeutic support units, 10 mobile units, 44 polyclinics, four hospitals, two Health Surveillance Units and one central laboratory [22].

### Study population

This comprised cases of leprosy (International Statistical Classification—ICD 10 from A30.0 to A30.9) that were diagnosed between 2003 and 2015 and residents of the urban area of the city.

### Study variables and data source

The study variables selected were diagnosis date, date of birth, sex, race/color, level of schooling, address, new case (yes or no), pregnancy, clinical form, operational classification (paucibacillary or multibacillary), number of skin injuries, and disability grade at diagnosis (0, I and II) and at cure (0, I and II). These variables were obtained through the Disease Notification Information System (SINAN).

Regarding the social determinants, data from the Demographic Census of Brazilian Institute of Geography and Statistics (IBGE) were used. These included general characteristics of the household and people, personal income, household income, color/race, age, gender, and level of schooling. Following the theoretical framework of Chaptini and Marshman [10], the social determinants were grouped into three dimensions:

a) Socioeconomic determinants: proportion of households with monthly income per capita, proportion of literate people and proportion of people according to race/color.
b) Environmental determinants: proportion of households according to the number of inhabitants and housing and basic sanitation conditions.
c) Gender determinant: proportion of households without a female resident.

### Data collection

Data were gathered from the Health Surveillance Department of the Health Bureau in Foz do Iguaçu, Brazil.

### Data analysis

Exploratory data analysis was performed using Statistica version 12.0, Software, calculating absolute and relative frequencies for the categorical variables, such as sex, schooling, race, operational classification, clinical form and degree of disability at diagnosis.

The continuous variables age and number of lesions were categorized according literature [4,8,9]. The number of cutaneous lesions were classified following the criteria defined by WHO [8], in other words up to five lesions the authors considered paucibacillary and more than five lesions were classified as multibacillary.

The incidence rate was estimated considering the number of cases in the population in the middle of year. This was multiplied by the time of observation (the 'risk period', 13 years in this case) [7]. The size of population was obtained from IBGE - https://www.ibge.gov.br/.

The technique of georeferencing new cases of leprosy by geographic coordinates, according to the address of the residence, was applied. In order to obtain geographic information latitude and longitude, Google Earth Pro open access software was used considering the UTM (Universal Transverse Mercator) projections for South America (SAD/69—South American 1969 UTM zone 20s).

After conversion of the database to CSV (Comma-separated values) format and its configuration, TerraView version 4.2.2 software was used for the geocoding itself, which corresponds to the linear interpolation of the complete address, to a point in the corresponding segment of the street. Cases with incomplete addresses were excluded from the study.

Thematic maps were produced through the digital mesh in the Shapefile extension of the census tracts provided by IBGE. ArcGis version 10.5 software was used for this analysis.

The relative risk (RR), or risk of illness due to leprosy, for each census tract was obtained through the Spatial Scan Statistic [23], using the discrete Poisson model, with age and sex as covariables through SatScan version 9.3 software. In this analysis it was defined a window of 50% of the population at risk with the statistical significance tested for 999 interactions.

The risk was estimated within a grouping divided by the estimated risk outside and within a given area, calculated based on the expression [24]:

$$RR=\frac{c/E[c]}{\left(C-c)/(E[C]-E[c\right]}=\frac{c/E[c]}{\left(C-c)/(C-E[c\right]}$$ (1)

Where, c is the number of cases observed within the cluster and C is the total number of cases in the data set *E[C] = C*, with the analysis being conditioned to a total number of observed cases. The thematic maps were developed in ArcGis version 10.5 software.

Investigation of the spatial dependence of the social determinants and of the risk of illness due to leprosy was carried out by calculating the Global Moran I Index, using GeoDa 1.6 software. This analysis shows the degree of spatial autocorrelation, where the values vary from -1 to 1; positive values indicate direct correlation and negative values inverse correlation, i.e., dissimilarity among neighbors; the value zero indicates that there is no spatial dependence. The Global Moran I Index was obtained using the formula [25]:

$$I=\frac{\sum_{i=1}^{n}\sum_{j=1}^{n}w_{ij}\left(z_{i}-\bar{z}\right)\left(z_{j}-\bar{z}\right)}{\sum_{i=1}^{n}{\left(z_{i}-\bar{z}\right)}^{2}}$$ (2)

With *n* representing the number of areas, *z*_*i*_ the value of the variable considered in the area *i*, *z* the mean value of the variable in the study region, *z*_*j*_ the value of the variable considered in the area *z*, and *w*_*ij*_ the neighborhood matrix (matrix by “queen” type contiguity). In this type of matrix, units with common boundaries or vertices are defined as neighbors, the neighboring unit being defined as *w*_*ij*_ = 1, while elements that have no neighborhood relation are defined as *w*_*ij*_ = 0 [25].

To test the association between social determinants and the risk of illness due to leprosy, the authors used bivariate Global Moran followed by multivariate analysis for the variable statistically significant in the first phase.

Calculations of the bivariate Moran index were performed using the GeoDa 1.6 software and the permutation of the neighboring attributes approach was used in evaluating significance, as described by Anselin [26].

In the multivariate analysis, the Ordinary Least Squares (OLS) regression was considered initially, in an attempt to explain the global relations between social determinants and risk. The diagnoses of an OLS model were followed by examination of multicollinearity and the residuals, quantifying the Variance Inflation Factor (VIF) values, and in cases where VIF was higher than 10, this indicated multicollinearity.

The GWR local model was used to analyze the relationship between social determinants and risk of illness due to leprosy for each area (census tract). This technique consisted of a localized multivariate regression that allows to make an estimate of the parameters of a regression and then how they have changed and ranged locally [27]. The adaptive kernel was chosen in this phase.

The adjusted coefficient of determination (Adjusted R^2^) and Akaike Information Criterion (AIC) obtained for OLS and corrected Akaike Information Criterion (AICc) calculated for GWR were used to compare the OLS and GWR models according to the procedure carried out by Lin and Wen (2011). All these analyses were performed using ESRI ArcGis 10.5.1 and R 3.4.2 software.

For the determination of the temporal trend, the annual incidence coefficient of leprosy was considered as the predictive variable (Y) and the time (year) the outcome variable (X). The annual incidence coefficient was converted into a logarithm, which provides the reduction of the heterogeneity of the residual variance of the linear regression analysis [28].

Temporal trend analysis was performed through the Prais-Winsten regression [28], based on the coefficients of annual detection of new cases of leprosy in the period studied. The confidence interval of 95% (95% CI) was used, which results in the Annual Rate of Increase (ARI) [29].

The rate trend was classified as increasing, stable or decreasing. In a rate with a positive value, the time series was considered to be increasing. A negative rate was considered to be decreasing and when there was no significant difference between the value and zero it was considered to be stationary. These analyses were performed using Stata version 13 *s*oftware. For all tests in the study, Type I error was set at 5% (p < 0.05) statistical significance.

### Ethical aspects

The Research Ethics Committee of EERP/USP, based on the Directives and Norms Regulating Research with Human Subjects, Resolution 196/96 of the National Health Council, approved the study under authorization No. 59299816.2.0000.5393 issued on 10^th^ September 2016. All data analyzed were anonymized.

## Results

### Epidemiological profile of cases

A total of 840 new cases of leprosy were identified, of which 427 (50.8%) were females, 685 (81.6%) were white race/color, 624 (74.3%) were people between 15 and 59 years of age, and 504 (60%) were those with incomplete high school education (Table 2).

**Table 2:** Demographic and clinical characteristics of the cases of leprosy in the tri-border region of Brazil, Paraguay and Argentina (2003 to 2015).

Variables	(n = 840)	%
**Sex**		
Male	413	49.2
Female	427	50.8
**Age**		
<15 years	29	3.4
15 to 59 years	624	74.3
≥ 60 years	187	22.3
**Education**		
Illiterate	112	13.3
Incomplete elementary education	504	60.0
Complete elementary education	18	2.1
Incomplete high school	92	11.0
Complete high school	22	2.6
Incomplete higher	6	0.7
Complete higher	19	2.3
No information	67	8.0
**Race**		
White	685	81.6
Black	53	6.3
Oriental	39	4.6
Brown	51	6.1
No information	12	1.4
**Operational Classification**		
Paucibacillary	192	22.8
Multibacillary	648	77.2
**Clinical form**		
Undetermined	109	13.0
Tuberculoid	77	9.2
Dimorphic	464	55.2
Lepromatous	183	21.8
Not classified/No information	7	0.8
**Number of cutaneous lesions**		
No lesions	55	6.5
≤ 5 lesions	441	52.5
5 lesions	342	40.7
No information	2	0.3
**Degree of disability at diagnosis**		
Grade 0	466	55.5
Grade I	125	14.9
Grade II	54	6.4
Not Evaluated	193	23.0
No information	2	0.2

Regarding the operational classification in Table 2, 648 cases (77.2%) were multibacillary and 192 (22.8%) were paucibacillary. The predominant clinical form was dimorphic (n = 464, 55.2%), followed by the lepromatous form (n = 183, 21.8%). With regard to the number of cutaneous lesions, 441 (52.5%) had five or fewer lesions and 342 (40.7%) had more than five cutaneous lesions, with a zero degree of disability (n = 466, 55.5%).

Of the 840 cases included in the study, 765 cases were geocoded (91%). Of the excluded cases, 50 did not have a full address and 25 cases were not located through the Google Earth Pro open access software.

### Social determinants and their relationship with the risk of leprosy

Table 3 presents the spatial statistics values obtained through the Univariate and Bivariate Global Moran I Test (BMI); following the theoretical framework of the study, it can be observed that the determinants were divided into socioeconomic, environmental and gender determinants. The Table 3 shows all variables were statistically associated with risk of illness due to leprosy.

**Table 3:** Univariate Global Moran I and Bivariate Global Moran I results of the social determinants and the risk of illness due to leprosy in the tri-border region of Brazil, Paraguay and Argentina.

Variable	Univariate Global Moran I	*p*-value (0.05)	Bivariate Global Moran I	*p*-value (0.05)
**Socioeconomic Determinants**				
Proportion of households with monthly nominal household income per capita of up to 1 minimum wage	0.7195	0.001*	0.1490	0.001*
Proportion of households with monthly nominal household income per capita greater than 1 minimum wage	0.7141	0.001*	-0.1477	0.001*
Proportion of literate people aged 5 years or more	0.1530	0.001*	-0.0504	0.002*
Proportion of people of white race	0.4383	0.001*	-0.0943	0.001*
Proportion of people of black race	0.2091	0.001*	0.0397	0.04*
Proportion of people of oriental race	0.1932	0.001*	-0.0974	0.001*
Proportion of people of brown race	0.4630	0.001*	0.1017	0.002*
Proportion of indigenous people	-0.0174	0.387	0.0976	0.005*
**Environmental Determinants**				
Proportion of households with 4 or more residents	0.2854	0.001*	-0.0703	0.005*
Proportion of households with 5 or more residents	0.2854	0.001*	0.0703	0.008*
Proportion of households with a bathroom or toilet for the exclusive use of the residents	0.0588	0.02*	-0.0743	0.013*
Proportion of households without a bathroom or toilet for the exclusive use of the residents	0.0588	0.03*	0.0743	0.025*
**Gender determinants**				
Proportion of households without a female resident	0.3556	0.001*	-0.0566	0.005*

*Statistically significant (*p* < 0.05)

Table 4 shows the results obtained from multivariate analysis applying Weighted OLS Regression, where the variables proportion of households with monthly nominal household income per capita greater than 1 minimum wage (β = 0.025, p = 0.036) and proportion of people of brown race (β = -0.101, p = 0.024) were statistically significant associated with risk of illness due to leprosy.

**Table 4:** Multivariate analysis from Weighted OLS regression of social determinants and their relationship with risk of Leprosy in the tri-border region of Brazil, Paraguay and Argentina.

Variable	Estimate	Std. Error	p-value
Intercept	6.433	1.922	0.001*
Proportion of households with monthly nominal household income per capita of up to 1 minimum wage	0.038	0.024	0.118
Proportion of households with monthly nominal household income per capita greater than 1 minimum wage	-0.101	0.045	0.024*
Proportion of literate people aged 5 years or more	-0.016	0.009	0.063
Proportion of people of brown race	0.025	0.012	0.036*

Multiple R-squared: 0.0942; Adjusted R-squared: 0.07978

Model p-value: < 0.001; AIC:1302.308

**Fig 2:**
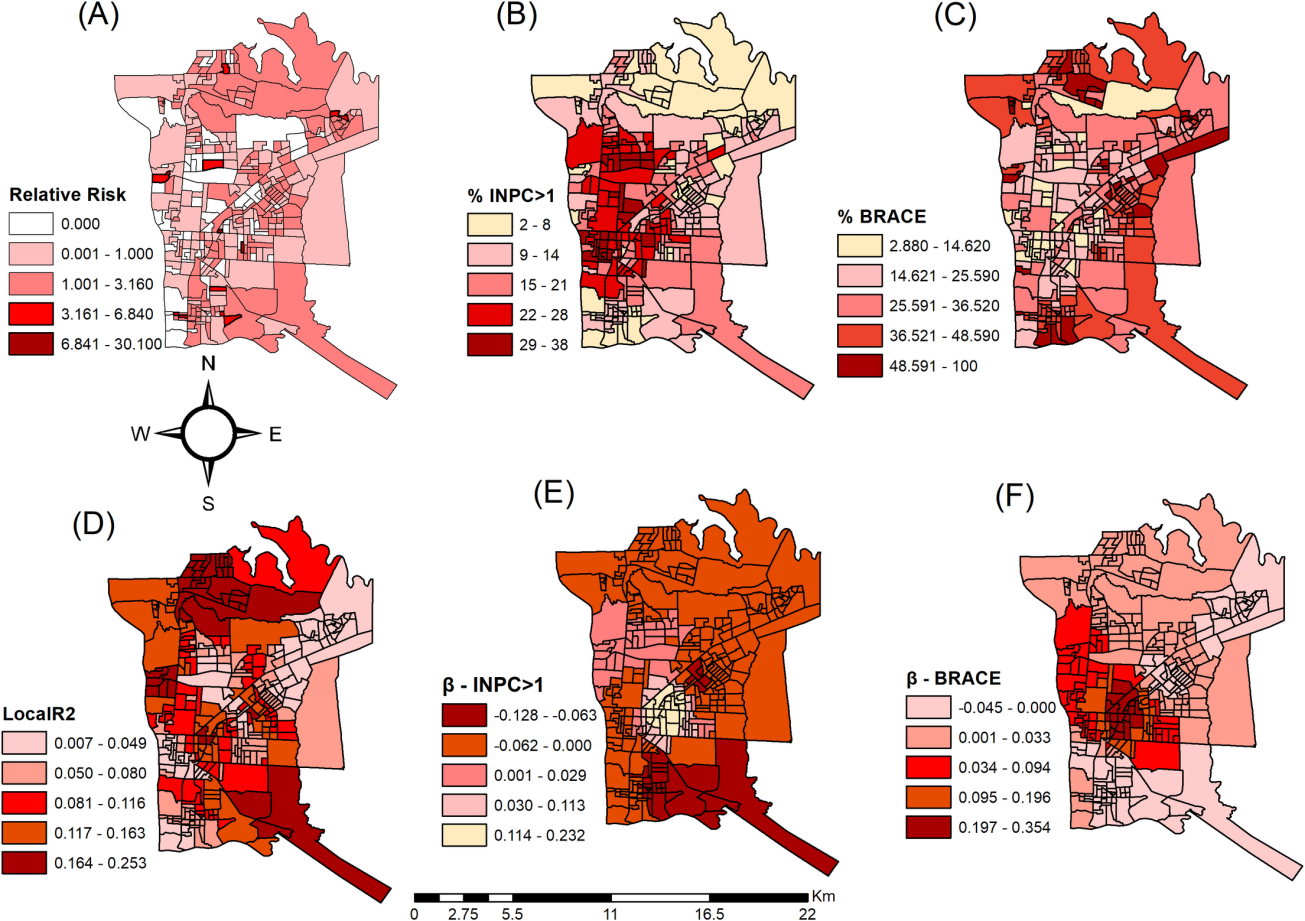
Spatial mapping by geographically weighted regression (GWR) of social determinants and their relationship with the risk of leprosy within the tri-border region of Brazil, Paraguay, and Argentina. (A) Relative Risk, (B) Proportion of households with monthly nominal household income per capita greater than 1 minimum wage (INPC > 1), (C) Proportion of people of brown race (BRACE), (D) R^2^ Adjusted, (E) Coefficients β of proportion of households with monthly nominal household income per capita greater than 1 minimum wage (INPC > 1), (F) Coefficients β of proportion of people of brown race (BRACE).

Fig 2 shows the maps obtained through GWR analysis. From this figure it is possible to observe the distribution of R^2^ and β of the variables. The results of GWR are really clear in the map and confirmed the GWR (R^2^ = 0.25; R^2^Adjusted = 0.16) was more suitable than the OLS model (R^2^ = 0.09; R^2^Adjusted = 0.08) since GWR explained 16 percent of the total model variation with the decreased AIC (1289.4).

Fig 2A shows the relative risk for tuberculosis, whereas the variables proportion of households with monthly nominal household income per capita greater than 1 minimum wage (%INPC > 1) and proportion of people of brown race (%BRACE) are shown respectively in Fig 2B and 2C.

The spatial variations in parameter estimates (β) for variables proportion of households with monthly nominal household income per capita greater than 1 minimum wage (βINPC > 1) and proportion of people of brown race (βBRACE) are shown respectively in Fig 2E and 2F.

Fig 2E demonstrates that negative coefficients values ranged from -0.12 to 0.23, from which it is evident that the risk is lower or nonexistent in areas where the income is higher than 1 minimum wage. Turning to Fig 2F, the parameters were from -0.04 to 0.35, which means there is a positive value in association of brown race to risk of illness due to leprosy.

### Temporal trend

Table 5 presents the results of the temporal trend, as revealed through the Prais-Winsten regression, in the annual detection rate of new cases of leprosy.

**Table 5:** Temporal trend of leprosy in in the tri-border region of Brazil, Paraguay and Argentina (2003 to 2015).

Variable	ARI (%)	*p*-value* (0.05)	95% CI	Trend
Rate of detection of new cases of leprosy	-4.3	0.000	-5.3, -3.3	Decreasing

ARI = Annual Rate of Increase (%); 95% CI = 95% confidence interval; Trend = interpretation of the trend

**Fig 3:**
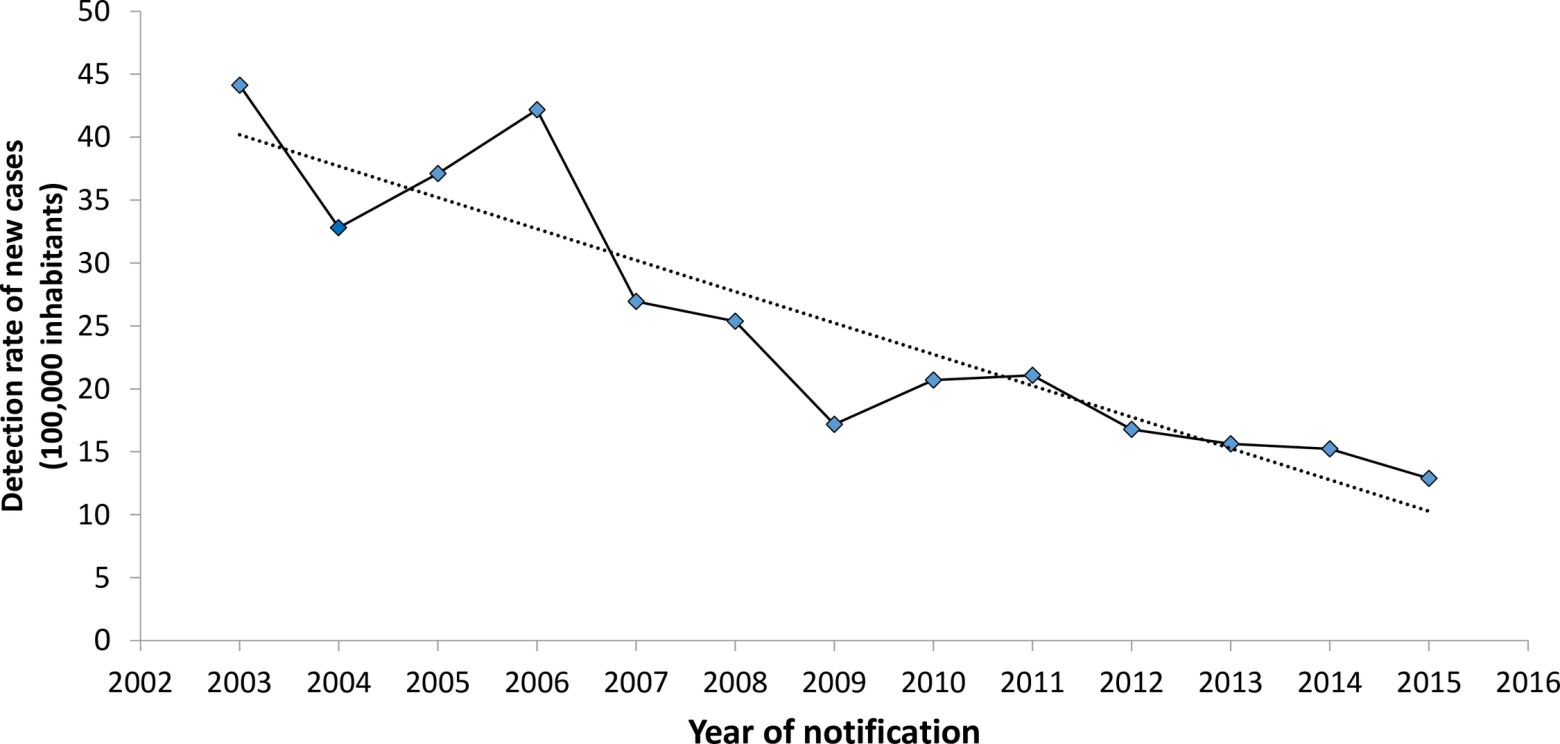
Temporal evolution of the detection rate of new cases of leprosy in the tri-border region of Brazil, Paraguay and Argentina (2003 to 2015).

Fig 3 shows a decrease in the detection rate of new cases of leprosy (4.3% per year) over the 13-year time series form 2003 to 2015.

## Discussion

The aim of this study was to analyze social determinants and their relationship with the risk of illness due to leprosy, as well as the temporal trends of leprosy cases in a region of the tri-border region in Latin America. Income and brown race were found to be determinants associated with the risk of leprosy. There was also a decreasing trend in the detection of leprosy in the region of the study, of the order of 4.3% in the period investigated, which was found to be significant, using the Prais-Winsten regression.

Initial exploratory analysis of the data revealed that there was a predominance of patients of white race/color, in the age group of 15 to 59 years and in those with incomplete elementary education. These results are similar to those found in other Brazilian studies [20,30–35].

Regarding age, the majority of the patients were in the economically-active age group, which is of concern, since the non-diagnosis of the disease can lead to the development of lesions and further evolve into disabilities and reactional states, which may impact on the family, and wider local, economy [9].

Patients under 15 years of age were also observed in the patient population (n = 29, 3.4%); this is a serious finding that indicates delayed leprosy diagnosis (high bacillary burden, without treatment) and a possible failure in primary care [5,8,12,36,37]. It is interesting to compare this result with Table 4, where the leprosy detection rate was identified as decreasing in the region since 2003.

Therefore it is likely that cases of disease are underreported, or not found, by the health care system, which might be due to a weakness of Primary Health Care in active case-finding and decentralization to general practitioners without support from a reference center for the diagnosis and treatment of leprosy [38].

This epidemiological situation is evidence that Brazil is really far from achieving the goal of WHO’s “Global Strategy for Leprosy 2016–2020”, which aims for zero children diagnosed with leprosy and visible deformities [8].

Also according to the results, most of the patients had incomplete elementary education, which is an important characteristic also found by Chaptini and Marshman [10,18,36] whose studies reported that low levels of literacy are associated with higher rates of leprosy. This relationship is due to a reduction in seeking and understanding clinical information regarding the disease and an association with low income.

Santos, Castro and Falqueto [39] also found that people with low levels of education have greater difficulty in accessing health services and understanding health promotion actions and disease prevention measures.

Regarding the operational classification of leprosy, the multibacillary form, the most infectious form of the disease, was observed predominantly. This may indicate late diagnosis of the disease in the study region, contributing to the leprosy transmission chain and to the increase in the degree of physical disabilities [2,9]. A prevalence of the multibacillary classification has also been reported in other studies [30,34,35,40,41].

Regarding the association between social determinants and risk of illness due to leprosy, it was possible to detect a negative association with the proportion of households with monthly nominal household income per capita greater than 1 minimum wage. This finding has been confirmed by other studies [24,42–46].

One important issue that has been shown by the literature is that income is a social protection factor in the development of neglected diseases that are related to poverty, such as leprosy [47]. It can clearly be observed in Fig 2C, where the relation between higher income and lower risk of leprosy is expressed, represented in the dark red color in the mapping.

Chaptini and Marshman [10] stated that leprosy is associated with poverty due to a multiplicity of factors, including the effect of lower income and unemployment which reduces access to health services to purchase necessary medicines, poorer housing, and overcrowding of homes. Another aspect that is linked to low income is access to adequate food, since poor nutrition is related to an increased risk of developing leprosy [12,48].

Through multivariate analysis it was found that areas with a predominance of people with brown race/color presented an increased risk for leprosy, which demonstrates the relationship of this disease with this social determinant. Castro et al. [49] also found a correlation between race/color and the risk of the occurrence of leprosy. Biologically there is no evidence that color/ race is a risk for the development of leprosy (36), however in Brazil these characteristics are more related to social inequality.

Due to reasons of history, people of brown race/color have less access to elementary school, high school and higher education; they have lower income when compared to white people and also fewer opportunities to participate in formal work, and to live in houses with basic sanitation [50]. However, there is a strong politics of “quota” (affirmative action) in Brazil to improve the access of these people in an attempt to give underprivileged people better chances of getting free higher education and thus access to better opportunities.

The politics of quota in Brazil addressed to color/race have changed in recent years, and class criteria have become more acceptable than race for reducing Brazil’s social and racial inequalities, however there is much discussion about the appropriate policy solutions for the problem [51]. This study may contribute with this debate once it is evidenced that areas with a higher proportion of brown race/color patients have a higher risk of leprosy.

It is important to highlight that the characteristics of the space where these people are living is what is associated to the leprosy risk, which means that is not necessarily these individuals are getting sick or have been affected by the disease. The association was found in the ecological level, a phenomenon that is classically known as ecological fallacy.

The results also show a decrease of 4% per year in the rate of detection of new cases of leprosy, which is similar throughout the entire state of Paraná [22,52]. This result leads to the question of whether the apparent decrease reflects the actual epidemiological situation of leprosy or possible underreporting. Considering the social inequalities that increase the risk of leprosy found in the study, the second hypothesis is more likely to be confirmed.

Because it is a border region, where there is a major flow of people between the three countries, the control of communicable diseases is highly complex. It is difficult to precisely determine the areas of risk due to the very dynamism of this population [20,54,55]. However, the study advances knowledge by highlighting the social determinants of the risk of leprosy, serving as a reference for future studies that include spatial dependence in their structure, notably those that test relationships between social determinants and diseases of poverty, such as leprosy.

The elimination of leprosy goes beyond merely knowing what the social determinants of leprosy are, and medical technology for diagnosis and treatment will only have an impact if there are significant advances in social areas. Although there have been improvements in social conditions in Brazil, due to large investment in government programs, this has not yet been enough to overcome the sanitary conditions where leprosy is spread.

Control of the disease includes the dimensions of sanitation, living conditions and income, as well as the inequalities that still exist among people of the brown race/color and lower income, as, according to the findings of this study, they are at higher risk of illness due to leprosy. Inclusion policies can be a measure to reduce the differences in risk.

Latin America achieved a detection rate of about 2.7 new cases per 100,000 inhabitants, taking second position in the world in terms of cases numbers. Brazil was included in the priority list for leprosy elimination, even though the number of cases detected has substantially decreased during the last decade. It is important to emphasize that in Latin America, Brazil presents the largest number of cases (25,218) followed by Paraguay (341) and Argentina (295) [56].

An issue is the migration between these countries likely influences in leprosy incidence and prevalence in the region studied, however it was not possible to estimate its impact due to data from Paraguay and Argentina were not available.

The study has shown that leprosy is spatially determined, this being relevant for studies that aim to understand the dynamics of the disease using more robust methodologies, mainly through GWR, which resulted in better models than traditional methods (OLS).

The study advances knowledge about the distribution of the disease in the tri-border region of Brazil, Paraguay and Argentina, presenting evidence for improvement in health policies for control of the disease.

Castro et al. [49] reinforce the associative nature of the disease and social inequality, demonstrating that leprosy is not limited to illness process individual, but essentially from social factors accumulated in the life course of a population, since intrauterine growth until later adult life, which is really complex to analyze in a single study.

Other issue is that leprosy is not limited to social stressor factors within a single generation but should intertwine biological and social transmission of risk across generations, which also requires more complex and dynamics approach.

For future study, it would be interesting to develop a prospective study and also including data from Paraguay and Argentina through a data collect in loco. The inclusion of data from both countries was not possible because all their registers were still handwritten, they do not use computerized systems for monitoring leprosy cases.

Based on the findings of the study, it can be concluded that the leprosy elimination strategy must transcend the technological apparatus of the health sector, which is often focused on diagnostic technology and treatment, and must include essential aspects of human development and welfare.

The study contains limitations related to the use of the secondary data obtained from SINAN, which may present data instability and incompleteness due to failures to complete the data in the notification form. Another limitation was the difficulty in selecting the variables related to social determinants, these were based on the literature [10,15,43,45,53] but the number of studies using such an approach for leprosy is still limited. The methodology also addresses the issue of areas identified as protection actually being areas of underreporting.
